# Minimally perturbing a gene regulatory network to avoid a disease phenotype: the glioma network as a test case

**DOI:** 10.1186/1752-0509-4-15

**Published:** 2010-02-25

**Authors:** Guy Karlebach, Ron Shamir

**Affiliations:** 1 Tel-Aviv University, Haim Levanon St., 69978, Tel-Aviv, Israel

## Abstract

**Background:**

Mathematical modeling of biological networks is an essential part of Systems Biology. Developing and using such models in order to understand gene regulatory networks is a major challenge.

**Results:**

We present an algorithm that determines the smallest perturbations required for manipulating the dynamics of a network formulated as a Petri net, in order to cause or avoid a specified phenotype. By modifying McMillan's unfolding algorithm, we handle partial knowledge and reduce computation cost. The methodology is demonstrated on a glioma network. Out of the single gene perturbations, activation of glutathione S-transferase P (GSTP1) gene was by far the most effective in blocking the cancer phenotype. Among pairs of perturbations, NFkB and TGF-β had the largest joint effect, in accordance with their role in the EMT process.

**Conclusion:**

Our method allows perturbation analysis of regulatory networks and can overcome incomplete information. It can help in identifying drug targets and in prioritizing perturbation experiments.

## Background

In contrast to the gene-centric approach, systems biology [1] emphasizes the importance of the interactions between different genes in determining the phenotype. Instead of asking "what is the role of gene A", the question becomes "what is the role of gene A in system B". The activity (or inactivity) of a gene is therefore not viewed as an isolated event, but assigned a meaning in the context in which it is active. An analogy from the sphere of computer science equates the genome to a database, and the system's dynamic behavior to the execution of a computer program that uses the database [2-4]. This paradigm shift has two major implications for the biomedical community. First, it complicates understanding cellular processes as each component must be considered with respect to its environment. Second, the fact that alternative phenotypes correspond to alternative dynamic behaviors of the system offers considerable advantages, because it is technically easier to influence the dynamics of a cellular network than to modify the information coded in the genome. Combining computational tools, which can help overcome the complexity of biological networks, with wet lab testing can spearhead system-oriented research. In this paper we present a method that was developed with this principle in mind. Focusing on gene regulatory networks, we develop a method to find minimal perturbations that change the network dynamics. By modifying established network analysis algorithms from the field of computer science, we are able to cope with some of the difficulties commonly associated with this objective.

An important tool for network analysis that will be used in this work is network perturbation. A common procedure in model analysis, it refers to applying a modification of the network and observing its resulting dynamic behavior. Knockout, knock-down or overexpression of a gene in the network are examples of possible perturbations. The exact type of perturbation varies with the model and the goals of the modeler. In some cases the motivation is to observe how single entities respond [5,6], while in others it is to determine network robustness [7] or change in the global state [8,9]. For example, Sridhar et al. [10] find enzymes whose inactivation eliminates compounds from a metabolic network. The implementation of a perturbation for our purposes is described in the Methods section.

A related concept in theoretical computer science is *Minimal Cut Sets *[11]. In reliability theory, network elements (e.g. edges) have a failure probability (e.g. an electronic component that has a chance for malfunction). A network is called *reliable *if a set of paths within it connect a given subset of vertices, and the joint probability of the paths is above a given threshold. A minimal cut set is the smallest set of elements whose removal from the network makes the network unreliable. Network reliability shares some important similarities with the concepts proposed in this work, as we also associate the existence of non-existence of network elements with probabilities. A main difference between the two approaches is that identification of minimal cuts sets is a method for analyzing a network via its structural properties. In contrast, our analysis will address the network dynamics and hence will be based on the concept of trajectories, as explained below.

Our first modeling choice will be to model the network's regulators as discrete entities, an approach that proved effective in previous genetic regulatory network (GRN) analyses [7,12-16]. This level of abstraction reduces the need of the modeler to provide fine details [17], while being detailed enough to capture the main features of the GRN dynamics and render them easier to analyze. In addition, the abstraction lends itself to the development of effective methods for incorporating uncertainty in the regulatory functions [18-22]. The *global state *of a network is defined as a vector whose entries are the local states of all the network's components. The network traverses from a certain global state to another in discrete time steps as a result of the activity of regulation functions. We assume that regulation functions act in an asynchronous manner: that is, that at each time step any regulation function can occur, provided its output changes the global state. A *trajectory *is a sequence of global states that the network can traverse in sequence.

Given a set of trajectories T and a set of global states S, S is called a ***phenotype ***of T if every trajectory in T visits only states of S. Similarly, S is called a ***prohibited phenotype ***of T if no trajectory in T reaches any state in S. We say that a network N has a ***phenotype ***S (***avoids a phenotype ***S) with respect to a global state g if the set S is a phenotype (prohibited phenotype) of the group of trajectories that the network generates starting from the initial state g. The following question can now be formulated: "How can the network dynamics be manipulated in order to generate or avoid a specific phenotype?" Answering this question has important practical implications, such as promoting the discovery of novel drug targets [23-25] or the design of synthetic biological systems [26,27]. Therefore, it is desirable to have a systematic way to answer the question for different networks under different conditions.

This is quite difficult, even under the simplified discrete model of GRNs: first, model dynamics can be highly complex, and second, experimental methods give only indirect clues about the network design. The second problem makes it difficult to construct models for networks that have not been extensively studied, especially when the number of participating entities is large. As for the complexity of network dynamics, consider the simple example of a network of n genes where each gene is regulated by some of the others. Assuming that a gene can be in one of two states, ON or OFF, the network can assume 2^n ^different global states. For ten genes, this results in over one thousand states. For twenty genes, there will be over a million states. Hence, it is possible that from some initial states the network will traverse an exponential number of states. Even this scenario is a simplification, because it assumes the network is known with perfect accuracy, which is seldom true. We will address these problems in the following sections.

In this study, given some initial states of the system and a desired phenotype, we will determine how a network should be perturbed in order to generate that phenotype, where a perturbation sets the level of one or more entities and thus changes the network's traversals between global states. In order to apply our algorithm efficiently to the Boolean model, we translate the network into a Petri net [28], and utilize McMillan's unfolding algorithm [29] to search the state space of a perturbed network. When the structure of the network is not fully known, we assign probabilities to alternative structures, redefine a phenotype probabilistically, and generalize our method to handle this case. To the best of our knowledge, this is the first method that integrates the trajectories of multiple alternative network structures, an important objective given the quality of current knowledge about biological networks. We demonstrate this methodology on the human glioma GRN.

## Results

### Algorithm

**Figure 1:**
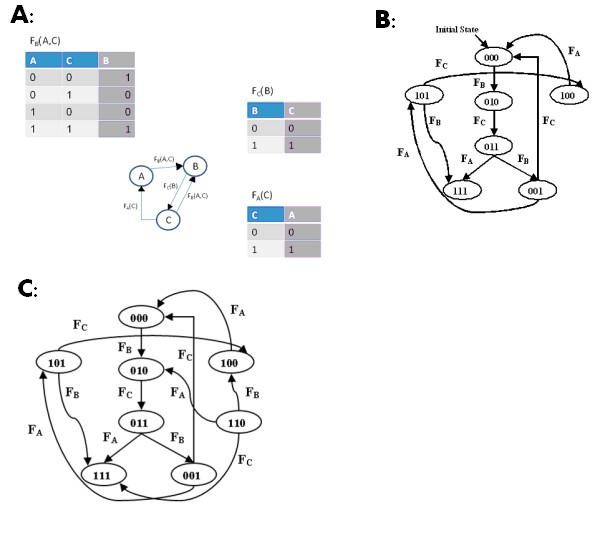
**A simple GRN and its state graph**. A: A simple GRN. The network contains three entities, A, B and C. Entities A and C regulate entity B, B regulates C, and C regulates A. Each table shows the level of the regulated gene when its regulation function acts, depending on the regulators' levels. B: The state graph of the GRN. Nodes correspond to global states (with coordinates A, B, C from left to right), and edges to transitions between these states. The labels on the edges show the regulation functions that cause this transition. C: The restricted state graph starting from the initial state 000. Only states that are reachable by transitions from 000 are shown. For simplicity, self loops are not shown. Sequences of state traversals that follow from the initial state can be cyclic (return to the same state) or can lead to the endpoint state 111.

Our model represents each gene by a distinct entity that can take one of two levels: level 1 means a gene is expressed and level 0 means it is not. The levels of genes are controlled by Boolean regulation functions, which can have any of the other genes (and even the gene itself) as inputs. The initial global state of the model is a vector that assigns an initial level to each gene. Starting from the initial state, the global state of the network can change in discrete time steps, where one regulation function is activated at each step. In other words, regulation functions can act in any order, and not all at the same time. This means that there can be more than one trajectory per initial global state. Figure 1A illustrates the model with a simple GRN.

We first give a description of our algorithm, followed by its implementation using Petri nets. The algorithm takes as input a network model, the network's state graph, a set of initial states A, and a group of states B. It outputs all the minimal perturbations that cause the network to have phenotype B with respect to every state in A.

Given a GRN N, its *state graph *is a directed graph G(V,E) whose nodes are global states of N. In G there is an edge (**a ,b**) if and only if there is a regulation function f that can act in state **a **and lead directly to state **b**. The label of (**a**,**b**) is the function f. Note that several labels are possible on the same edge if it is a self loop. Figure 1 illustrates a simple GRN and its state graph.

We define two operations on a network: An ***activation ***of a gene causes the gene to stay fixed on level 1. For example, if we activate gene B in Figure 1C, the network dynamics will lead to the endpoint state 111. Similarly, a ***repression ***of a gene causes the gene to stay fixed on level 0. In Figure 1C, repressing gene A will result in cyclic behavior that will lead back to the initial state. Self loops in the state graph are meaningless under these definitions, and therefore are omitted.

The biological means of activation and repression vary depending on the mechanisms of regulation [30-32]. Common examples are knock-down, overexpression, and addition of inhibitors and activators, but less standard examples can be thought of, such as insertion of artificial entities [33] or de-novo network design [26].

A ***network perturbation ***is a set of operations (activations and/or repressions) on genes. The maximal allowed size k of a perturbation P is assumed to be a small constant. An edge in the state graph ***contradicts ***perturbation P if it leads to a state in which an activated gene is at level 0 or a repressed gene is at level 1.

Let A and B be two groups of states in the state graph G, such that *A *⊆ *B*. We want to find a minimal perturbation such that the network has phenotype B with respect to every state of A. Assuming that k is constant, the following algorithm runs in time polynomial in the size of G

1. For i = 0,...,k do

For every possible perturbation set P of size i, do

i. Modify the group A according to P; i.e. set the level of activated genes to 1 and the level of repressed genes to 0.

ii. Add a node s and connect it by outgoing unlabeled edges to all the nodes of group A.

iii. Add a node t and connect each node of group  to it using outgoing unlabeled edges.

iv. Create a modified graph G' from G by removing all edges that contradict operations in P.

v. If there is no path from s to t, output the set P and stop.

2. If this step is reached, then there is no solution of size ≤k.

If one is interested in all the minimum solutions, then instead of halting after finding the first good perturbation of size i, halt only after enumerating all perturbations of size i. If B is a prohibited phenotype then step 1a(iii) should be changed: the node t should be connected to B instead of .

The running time on a state graph G = (V,E) is O(2^k^‧n^k^‧(|V|+|E|)), where n is the number of entities in the GRN: the creation of G' and searching for paths in it can be accomplished by a BFS, and the loop occurs O(2^k^n^k^) times. Hence this algorithm is practical if we assume that G is not too large. However, since |V| = 2^n^, only very modest sized GRNs can be directly solved this way in practice.

To address this complexity problem, we will formulate our problem using Petri nets and present a methodology that copes better with the state explosion problem.

**Figure 2:**
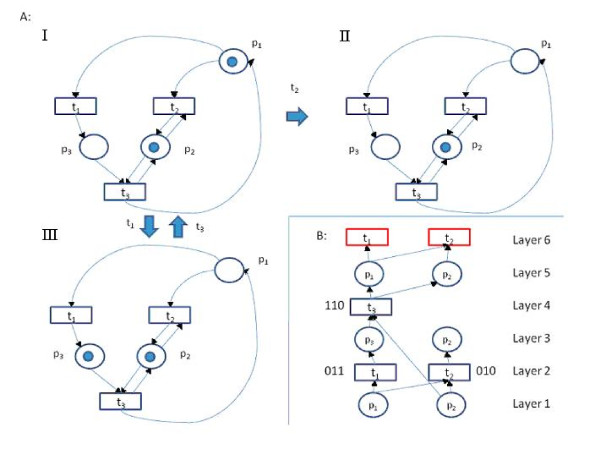
**A Petri net and its unfolding**. A: A Petri net and its unfolding. The net contains 'places' (light blue circles), the model's entities, and 'transitions' (rectangles), which constitute the regulation functions and define the model's dynamics. Arcs connect input places to transitions, and transitions to their output places. Places that receive discrete values are called tokens (blue dots). A transition that is activated, or 'fired', reduces the tokens in its input places and increases the number of tokens in each of its output places. At any time step, every transition that has enough tokens in its input places may be fired. In the example, every transition consumes one token from every input place, and produces one token at every output place. Labels next to thick arrows indicate which transition fired. Transitions t1 and t3 can be fired in alternation indefinitely, whereas no other transition can be fired after t2 has fired. B: Unfolding of the Petri net. Transitions are represented by rectangles, places by circles. The two places p_1 _and p_2 _that have tokens in the initial marking in state I are the input-less places of the unfolding. The local configuration of t_2 _at layer 2 corresponds to the marking 010, i.e. the marking in which only p_2 _contains a token, corresponding to II in **Figure 2A**. The local configuration of t_3 _corresponds to the firing of t_1 _followed by t_3_, and to the marking 110, i.e. the initial marking. The instances of t_1 _and of t_2 _at layer 6 are cutoff points, since their local configurations' markings are already represented by other local configurations. The graph constitutes a branching process.

Petri nets are a modeling formalism that has been used to model different types of biological networks [34-40]. A Petri net is a bipartite graph composed of two sets of nodes: *places *and *transitions *(see Figure 2A). The transitions set contains nodes that represent discrete events that can occur concurrently. The places set represents network entities. Transitions and places are connected by directed edges that represent interactions between network entities. The places having an edge into (from) a transition are called its preset (postset) places. The global state of the network is given by a discrete assignment of tokens to different places (the level of each entity), and is referred to as ***marking***. For example, the network in Figure 2A has three places, and the marking in I assigns one token to each of the place p_1 _and p_2 _and zero tokens to p_3_. Tokens can be consumed and produced by transitions. The rule that determines token consumption and production is called the ***firing rule***, and it allows a transition to fire (consume and produce tokens) if every one of its preset places contains a specified amount of tokens. When fired, a transition consumes these tokens and produces a set number of tokens to every one of its postset places. See Figure 2A for an example.

Reddy et al [41] introduced the use of Petri nets in the context of systems biology. Later, Chaouiya et al. [42] suggested a methodology for translating Boolean regulatory networks into Petri nets, which we adopt. Additional examples of modeling GRNs with Petri nets are refs. [43-45]. Translating the network to this framework has the advantage of a rich literature on techniques for analyzing the dynamics of Petri nets. In addition, Petri nets are suitable for describing other types of biological networks, such as GRN models with additional metabolic and signaling layers.

McMillan's unfolding algorithm [46] is a method for dealing with the state explosion problem for Petri nets. A full description of the unfolding algorithm can be found in ref. [47]. Briefly, given an initial state, McMillan's algorithm gradually and implicitly records the states reachable from it by constructing a directed graph called a branching process. A ***branching process graph ***begins with places that correspond to the initial marking of the Petri net, and transitions that are added to it can consume from these places and produce new places, thereby representing consumption and production of tokens. A transition can consume only from places that do not belong to conflicting firing sequences, i.e. firing sequences that cannot occur concurrently. Thus, additions of new transitions preserve the acyclic property of the branching process graph, and ensure that it represents only feasible firing sequences (Figure 2B). Refs. [46,47] provide excellent illustrations of the algorithm's capacity to reduce the search space on larger network instances.

Every reachable marking has a subset of transitions in the branching process graph that correspond to the firing sequence that generates it. These subsets are called ***configurations***. For a transition t, the set of transitions from which there is a directed path to t is referred to as t's ***local configuration ***(denoted [t]), and is associated with a marking. The marking of [t] is the marking obtained by firing all the transitions that belong to [t].

**Figure 3:**
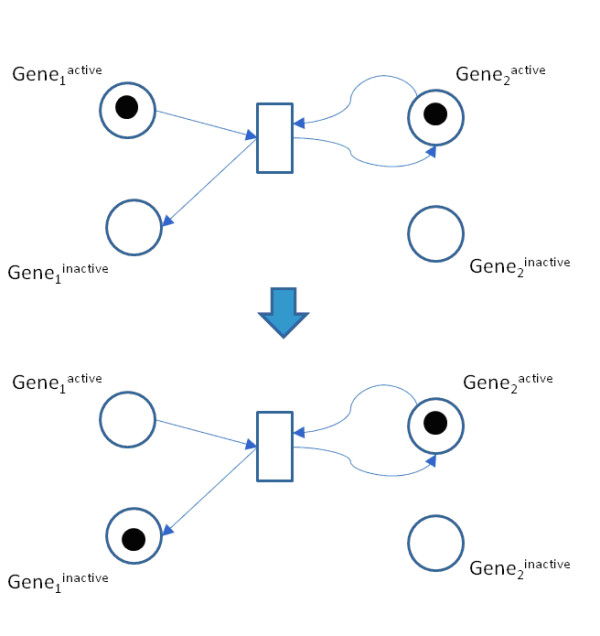
**Petri net representation of a Boolean entity**. In this example gene 2 inactivates gene 1. Each of them is represented by two places in the Petri net. The upper (lower) part of the figure shows the Petri net before (after) the transition fires. When gene 2 is active, it inactivates gene 1. Therefore, the transition consumes a token from the active place of gene 1, and produces a token to its inactive place. The transition also consumes a token from the active place of gene 2, and produces a token to the same place. The latter consumption and production express the fact that gene 2 needs to be active in order to inactivate gene 1, but the inactivation itself does not change the level of gene 2.

In the GRN representation that we adopted, every entity e corresponds to two places: one represents its active level and the other represents its inactive level. The firing rules are set so that exactly one of the places is marked at any time, i.e. each pair is place invariant ^37^. These places will be called the active and inactive places of the entity e. Figure 3 illustrates this concept.

The unfolding algorithm can produce a much smaller graph than the complete state graph. The following preprocessing to the algorithm spans all the states that are reachable from a given initial state under a perturbation P:

1. For every activated entity e in P, set a token in the active place of e.

2. For every repressed entity e in P, set a token in the inactive place of e.

3. Remove all transitions that have edges outgoing to places contradicting P.

When there are several initial states, a branching process graph is generated for each initial state.

The above algorithm requires full information about the GRN model. Since this is usually not the case, we now address the handling of ambiguities in the GRN logic. Consider a network in which every gene can have several alternative regulation functions, each associated with a probability that it is the true regulation. The events corresponding to the true regulations of different genes are assumed to be independent. Hence, the probability of a trajectory is the product of the probabilities of the regulation functions involved in it. Given a parameter α, 0 ≤ α ≤ 1, the definition of a phenotype can now be extended as follows: A network has a phenotype P with respect to a set of initial states if every subset S of regulation functions that has probability ≥α generates only trajectories that remain in P. Note that if the condition holds for S it will hold also for every *S' *⊆ *S*, which can have higher probability. This definition induces a distribution of all alternative networks into layers. The top layer contains networks with probability ≥α. Sets of networks with lower probabilities belong to lower layers, each layer corresponding to a different probability. The lowest layer has probability α^N^, where N is the number of entities. Higher layers have lower capacity because there can be less networks with high probability than networks with low probability (as all probabilities must sum to 1). For networks in the top layer we examine every possible trajectory - this follows from the definition of probabilistic phenotype, since the full set of regulation functions of these networks has probability ≥ α. As we descend in the hierarchy, layers have greater capacities and contain networks of lower probabilities. For every such network we examine only trajectories that are generated by strict subsets of their regulation functions, because the full sets of regulation functions of these networks have probability <α. In other words, in lower layers we still follow the dynamics of every network, but to a lesser extent than in higher layers, and so each structure has an influence on the phenotype in proportion to its probability.

Similarly, a network has a prohibited phenotype P with respect to a set of initial states if every subset S of regulation functions that has probability ≥α does not generate any trajectory that leads to P.

A naïve way to test for a probabilistic phenotype would be to repeat the non-probabilistic algorithm for every set of regulation functions with probability >α. However, the number of such sets grows exponentially with the number of entities that have more than one regulation function. More specifically, assume that there are n genes and every gene has k alternative regulation functions. For each gene, a set can specify one of the k regulation functions or leave that gene unregulated, i.e. not commit to a specific function. This gives rise to (k+1)^n ^alternative sets of regulation functions. If k is constant, the expression is exponential in n. Next we discuss how to modify the unfolding algorithm to test for a probabilistic phenotype.

Since we translate a regulatory network into a Petri net, every transition of a configuration C in the branching process graph corresponds to a regulation function (recall that in the probabilistic setting, one gene may have several regulation functions). Denote by φ(C) the set of regulation functions that are represented by the transitions of C. Note that if C contains a single regulation function for each entity, the size of φ(C) is at most the number of entities in the model. Denote by φ'(C) the subset of φ(C) that contains only regulation functions with probability <1.0. We say that φ'(C) is ***unambiguous ***if it does not contain two regulation functions that regulate the same entity.

A key concept in the original unfolding algorithm is a cutoff point; it is a transition t whose local configuration [t] is associated with a marking that is also associated with some other local configuration [t'] that contains fewer transitions. At cutoff points one can prune redundant branches in the constructed branching process graph. Given such a pair of transitions t and t', we modify McMillan's cutoff criterion to handle probabilities by adding another condition that must hold for t to become a cutoff point:

***Cutoff criterion 1: **φ'*([*t'*]) ⊆ *φ'*([*t*])

In addition, we make sure that each local configuration is unambiguous by keeping track of the functions that have been utilized in it, and allowing a transition t to fire from C only if φ'(*C *∪ {t}) is unambiguous.

Finally, in order to save time and space, we add another cutoff criterion to the algorithm

*Cutoff criterion 2:*

A transition is a cutoff point if the product of the probabilities of regulation functions that are used in its local configuration is <α.

Note that since we tightened the cutoff criterion, the size of the branching process graph can become larger than in McMillan's algorithm.

Theorem 1: The modified version of McMillan's algorithm maintains:

1. For a phenotype P: If there is a set of regulation functions F with probability ≥α that generates a trajectory that does not remain in P, then such a trajectory will be represented by a configuration C in the branching process graph and *φ'(C) *⊆ *F*.

2. For an avoided phenotype P: If there is a set of regulation functions F with probability ≥α that generates a trajectory that leads to P, then such a trajectory will be represented by a configuration C in the branching process graph and *φ'(C) *⊆ *F*.

The proof is provided in the Appendix. Given that the theorem holds, we simply need to construct the branching process graph and test for such a configuration C in order to verify that a phenotype is maintained or avoided.

### A Test Case

Shmulevich et al [48] constructed a probabilistic model of a small autonomous subnetwork of genes based on human glioma gene expression data [49] obtained for 588 known genes, in tissue samples with differing levels of glioma severity. The inferred network was used for a Probabilistic Boolean Network (PBN) simulation [50] by Akutsu et al. (The probability of a regulation function is the sum of coefficients of determination (CODs) between expression levels of each of its input genes and the output gene divided by the sum of CODs of the expression level of the output gene and all its potential regulators [48].) In view of its intriguing dynamic behavior and biomedical relevance, we used that network model to test our minimum perturbation set algorithm. After removing a gene that had no regulators, 14 entities remained, each associated with 1-3 regulation functions. When there is more than one function for an entity, the functions are assigned probabilities that add up to one. Six genes have a single regulation function, seven genes have two alternative regulation functions, and one gene has three possible regulation functions. A description of the logic functions appears in ref. [50]

**Figure 4:**
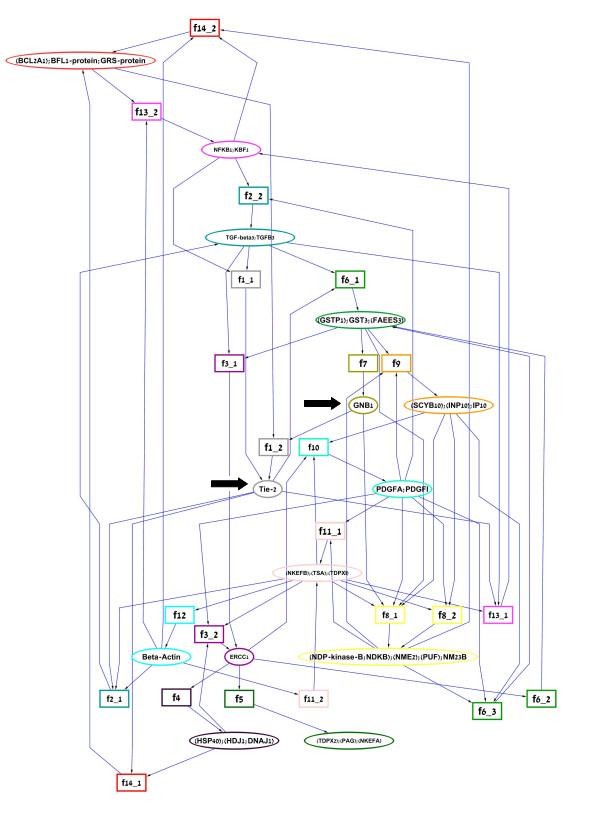
**The glioma network**. Genes (ovals) and their alternative regulation functions (rectangles) are bordered by frames of the same color. Ovals contain the name of the relevant human gene, following the nomenclature in [48]. Rectangles contain the name of the regulation function [49]. Regulation functions are connected by directed edges to the gene they regulate. Regulators are connected by directed edges to the regulation functions in which they are involved. The figure was generated using Cytoscape [59]. The bold arrows indicate the two entities that constitute the prohibited phenotype (see text).

We transformed this network into a Petri net (Figure 4), and applied our algorithm to find minimum perturbations from 1000 random initial states. The initial states were tested in this way because the "biologically correct" initial states cannot be derived from current knowledge. Moreover, since the glioma network is manifested in dividing cells that constantly redistribute their molecular contents, it is not unrealistic to assume a variety of initial states.

We defined the prohibited phenotype S of the network as where as the set of global states in which the gene Tie-2 for the receptor Tie-2[51] and the gene GNB1 for the human G-protein beta subunit [52] are both expressed (see Figure 4). The set S was selected following reports that vasculogenesis, an important phase in tumor progression, is initiated by a signal to the receptor Tie-2 that is propagated through a G protein [53,54]. Since repression of either Tie-2 or GNB1 is a trivial solution, these genes were excluded from the perturbations tested. Similarly, initial states in which both Tie-2 and GNB1 are active were excluded from the set of possible initial states, because there is trivially no solution from these states. The parameter α, which determines the least probability of a trajectory that will be explored - and hence the running time of the algorithm, was set to 0.05.

**Figure 5:**
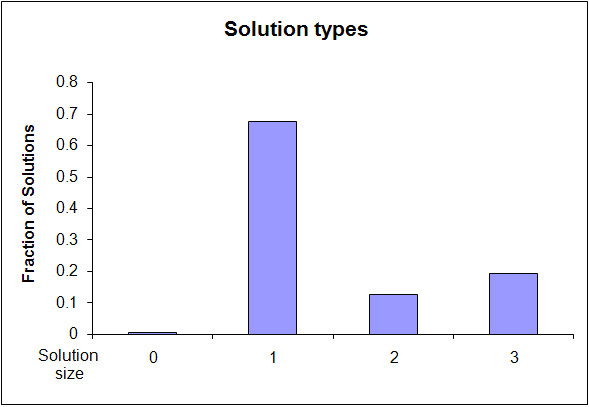
**Frequency of perturbation size needed**. The histogram plots the fraction of solutions of each size. "Size 0" indicates states from which the avoided phenotype is not reachable.

Figure 5 shows the distribution of solution sizes found. In about 0.5% of the initial states the phenotype is avoided without any perturbation. Perturbations of size 1 cause the network to avoid the phenotype in about 65% of the initial states, and perturbations of size 2 and 3 are needed in the remaining cases.

**Figure 6:**
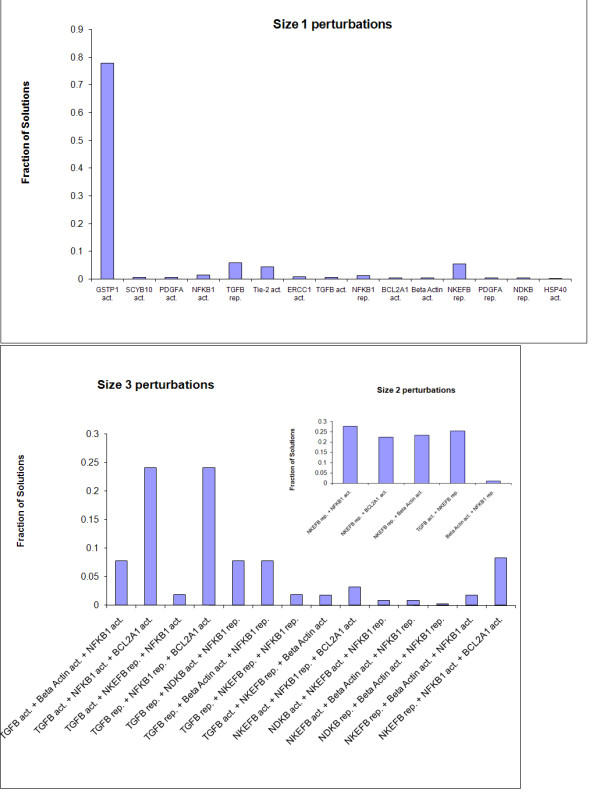
**Frequency of minimal perturbations of sizes 1, 2 and 3**. Each bar shows the proportion of the occurrences of a different perturbation. Act: activation; rep: repression

Figure 6 shows the frequency of perturbations of different sizes. It should be pointed out that when there are several perturbations of the minimal size, all of them are found. As can be seen in the figure, the number of perturbations that provide minimal solutions is much smaller than the total number of possible perturbations. The activation of the gene GSTP is by far the most abundant operation in size 1 perturbations. The probability that all the operations that appear at least once in size 1 perturbations are equally likely is 0.0001 (Χ^2 ^test, 14 degrees of freedom). In addition, in contrast to other genes, GSTP is only activated and never repressed. Reassuringly, these facts are consistent with experimental observations [55]

• Mice deficient in GSTP are viable, fertile, with life spans essentially similar to animals not deficient in the gene. However, they show an enhanced susceptibility to carcinogen-induced skin papillomas.

• The absence of GSTP increases the activity of stress kinases, which results in changes in gene expression that enhance cell proliferation pathways.

• Hypermethylation of the GSTP regulatory region is a common somatic alteration identified in human prostate cancer. This alteration results in the loss of GSTP expression and is proposed to occur during pathogenesis of the disease.

• In the latter case it was suggested that there could be therapeutic value in restoring GSTP activity, although it has not been tried.

Our results are consistent with these observations. They single out the activation of GSTP as an operation that blocks tumor progression.

In initial states where no size 1 perturbation suffices, GSTP does not participate in a perturbation. This is consistent with the observation that GSTP is often highly expressed in cells that have already turned malignant.

There are four common perturbations of size 2. All of them include repression of natural killer enhancing factor B, accompanied by activation of one of BCL2A, TGF-β, NFκB, or Beta-Actin. The first two operations are associated with repression tumor cell death, while the latter three are associated with constant induction of cell migration.

The most common perturbations of size 3 are activation of both TGF-β and NFκB or repression of these entities in addition to activation of the entity BCL2A1.

These results can be understood in the context of the stages of glioma progression Zagzag et al. distinguish three stages that precede the formation of new blood vessels.

a. In the first stage tumor cells migrate and adhere to existing blood vessels. Huber et al. concluded that NFκB, at least in part, substitutes for TGF-β in the process of EMT, which is essential for tumor migration.

b. In the second stage of tumor progression, blood vessel cells undergo apoptosis, and the nearby tumor cells undergo necrosis. Breaking cell-to-cell adhesion is thought to be a trigger for the apoptotic process. Disrupting cell migration or preventing apoptosis may halt the regulatory program at the second stage.

c. In the final stage, new blood vessels are formed. The initial states that correspond to this stage are included in the prohibited phenotype.

Thus, the combination of anti-apoptotic signals in addition to setting of cell migration signals in size 2 and size 3 perturbations may correspond to blocking of apoptosis and disrupting the formation of blood vessels, and halting the regulatory program at the second stage. The most common size 1 perturbation may correspond to prevention of the first stage of tumor progression.

We interpret our findings in light of existing experimental data as follows: GSTP can prevent the initiation of the vasculogenesis program. In later stages it is no longer effective, but other genes can be disrupted in order to halt this program, depending on the stage of vasculogenesis - the later the stage the larger the perturbation that is needed.

All executions were performed on x86 64 bits machines with Pentium IV or Zeon processor and at most 2 GB RAM. Jobs were run in a time-sharing environment and therefore the running times are only an upper bound. The program code was written in C. After 12 hours, 70% of the jobs finished. Since the rest of the jobs required more than 24 hours, we used only those 70% that concluded early in our analysis.

## Discussion

System-level analysis presents researchers with new challenges and at the same time offers new opportunities for better understanding of the biology. The complexity of reconstructing biological networks and analyzing their dynamics makes computational tools essential for system-level approaches [56,57]

We have described a computational method that determines the minimum size perturbations required for obtaining (or avoiding) a specific phenotype. Because the function of genes depends on the global context in which they are active - the state of the system - the phenotype cannot be represented by the activity or inactivity of a single gene, but rather by the global state of the network. We therefore defined a phenotype based on network dynamics as a set of global states that must be preserved (or avoided), and designed an algorithm that follows this definition. The method was implemented for a probabilistic Boolean model, and was demonstrated on a glioma network.

We showed that two major problems in network analysis, namely state explosion and partial knowledge, can be alleviated by translation to Petri nets and extensions of the unfolding technique. Our method demonstrates the power of computational analysis of the network's dynamics. On the glioma network it singled out one perturbation of size 1 whose effect on the phenotype was strongest. That perturbation has strong support in the literature. In addition, the most prominent perturbations of sizes 2 and 3 can be explained in the context of glioma progression. We expect this method can be used to derive such insights for other networks, because it does not require perfect knowledge and uses the broadly applicable Petri net semantics.

Though the paper focuses on GRNs, the suggested computational method can be applied to signaling or metabolic networks and to networks that integrate several layers, e.g. metabolic and regulatory. Petri nets have been used for modeling all these network types.

Our method has several limitations: Some instances of the problem still require exponential running time, making our method impractical for finding a minimal perturbation for large models. Our method is sensitive to modeling accuracy and depends on the correctness of prior knowledge, albeit in a probabilistic setting. In addition, we assume that the network is asynchronous, while in some cases the order of occurrence of regulation functions may be determined by large rate differences among them.

Improving the algorithm's performance is one of our future goals. The minimal perturbation algorithm can be used in practice only when the size of a perturbation is small; allowing larger perturbations requires new algorithmic ideas. However, to date it is impractical to perturb more than a few entities in the cell, making speed-ups useful primarily for analyzing larger networks. The case where some of the entities are synchronized and some are not can also be considered (Ref. [37] shows how synchronized networks can be modeled with Petri nets). Finally, the unfolding algorithm may be improved by modifying the cutoff criterion.

Other model checking techniques for Petri nets are described in ref. [58]. Though not directly related to unfolding, they provide alternative attempts to battle the state explosion problem when using the Petri net semantic.

## Conclusion

The ability to effectively manipulate a given network's dynamics in order to produce a desired behavior depends both on advances in experimental techniques and on the ability to computationally analyze the network. We presented a computational methodology for determining a minimum size perturbation yielding a desired phenotype that copes with some of the urgent difficulties in modeling. Application of this methodology to ongoing experimental projects and extension of its theoretical foundations are among our future goals.

## Authors' contributions

The authors designed and developed the method together. GK implemented the algorithm and carried out the testing. Both authors wrote the manuscript.

## Appendix

### Theorem

Let P be a phenotype (respectively, a prohibited phenotype). If there is a set of regulation functions F with probability ≥α that generates a trajectory that does not remain in P (respectively, that leads to P), such a trajectory will be represented by a configuration C in the branching process graph and *φ'(C) *⊆ *F*.

### Proof of the theorem

We prove the theorem for a phenotype. The proof for a prohibited phenotype is symmetric.

Let S be a state that does not belong to the phenotype, and let F be a set of regulation functions with probability ≥α such that F generates some trajectory that reaches S.

The proof is by induction on the number of state traversals (edges) in the state graph that are needed for reaching the state S. For purposes of the proof, we will use the term "infinite branching process graph" for a branching process graph in which cutoff points are not applied, and the term "finite branching process graph" for the branching process graph that is created by the algorithm.

### Base

Zero state traversals, i.e. the initial state. The initial state is reachable by every set of regulation functions. In the branching process graph it is represented by the initial marking. The set φ of the initial marking is the empty set, and therefore the theorem holds for the base case.

### Assumption

Every state that is reachable by a set of regulation functions with probability ≥α and N-1 edge traversals is represented in the branching process graph.

### Step

Let π be the path in the state graph that leads to S, and let N be the length (number of state traversals) in π. We want to show that some trajectory leading to S that is generated by a set of regulation functions with probability ≥α is represented in the branching process graph.

Let e be the last edge (state traversal) in π. The resulting path π' = π/{e} ends at some state S' and is of length N-1. Since there is a path of length N-1 to S' whose functions belong to the set F, by the inductive hypothesis S' is represented by some configuration C' in the branching process graph and *φ'(C') *⊆ *F*.

Let t' be the transition that represents the edge e. We want to show that t' can be added to the branching process graph to yield a configuration C that represents π.

First, all of the input places that t' requires are output places of C', and therefore are not in conflict. Add t' to the branching process graph such that it consumes from these places. Since *φ'(C') *∪ {*t' *}⊆ *F*, t' will not be set as a cutoff point according to cutoff criterion 2. If t' is not set as a cutoff point due to cutoff criterion 1 either, then we are finished, because we obtained a configuration C that represents S. Therefore assume that t' is set as a cutoff point according to cutoff criterion 1.

In the latter case, [t'] is already represented by some local configuration C''. According to cutoff criterion 1, *φ'(C") *⊆ *φ'([t']) *⊆ *F*. if *φ(C'') *⊄ *F*, i.e. there are some regulation functions with probability 1.0 that are used in C'' and not in [t'], then we will build the configuration C for a set F' that has the same probability as F, i.e. probability ≥α. Otherwise, we will build C for the set F. In any case we will have *φ'(C') *⊆ *F*.

Now, note that if [t'] was not set as a cutoff point, then the set of transitions C/[t'] could have been added to [t'] in the branching process graph to yield the configuration C. Intuitively, imagine that the branching process graph is infinite, i.e. cutoff points are not used at all. Since C'' corresponds to the same marking as [t'], transitions that correspond to the transitions of C/[t'] in the original Petri net can be added to C'' in an infinite branching process graph to produce a new configuration C''' that is smaller than C and *φ'(C''') *⊆ *F*. If the new configuration is not represented in the finite branching process graph, then it must also contain a cutoff point t''. We can repeat the same process with the cutoff point t'', until we get a configuration that has the same marking as C, uses only functions of F (or of a set with equal probability), and is represented in the finite branching process graph. We will surely obtain such a configuration because each time that we repeat this process the local configuration that corresponds to the cutoff point becomes smaller, and the minimal size of a configuration is 0.
